# Interpretable machine learning for predicting moderate-to-severe insomnia risk in Chinese adults with autism spectrum disorder

**DOI:** 10.3389/fpsyt.2026.1898010

**Published:** 2026-09-15

**Authors:** Zhenhao Lin, Yuwen ShangGuan, Young-Je Sim, Xue Jia, Xianxian Deng, Yunhan Qing, Yongyu Huang, Yisong Yao, Wenle Zhang

**Affiliations:** 1 Department of Exercise Physiology, Kunsan National University, Gunsan, Republic of Korea; 2 School of Rehabilitation Medicine, Jining Medical University, Jining, Shandong, China; 3 The Primary Health Development Research Center of Sichuan Province, North Sichuan Medical College, Nanchong, China; 4 School of Physical Education and Sport Science, South China Normal University, Guangzhou, China; 5 Chinese Evidence-Based Medicine Center, West China Hospital, Sichuan University, Chengdu, China; 6 Department of Psychological Assessment, Xiamen Xian-Yue Hospital, Xian-Yue Hospital Affiliated with Xiamen Medical College, Fujian Psychiatric Center, Fujian Clinical Research Center for Mental Disorders, Xiamen, China

**Keywords:** autism spectrum disorder, machine learning, moderate-to-severe insomnia, risk prediction, SHAP

## Abstract

**Objective:**

To develop a risk prediction model for moderate-to-severe insomnia among adults with autism spectrum disorder (ASD) and to identify key predictive features using interpretable machine learning methods, to support exploratory risk estimation and stratification.

**Methods:**

This study used data from the 2024 Psychological and Behavioral Survey Database of Adults with ASD and included 976 adults with ASD in the final analysis. Moderate-to-severe insomnia was defined as an Insomnia Severity Index (ISI) score ≥ 15. To avoid outcome leakage, all ISI items and the ISI total score were excluded from the predictor set, and sleep-related items were also removed from the depression and anxiety symptom scores. The dataset was first divided into training and test sets at a ratio of 7:3 using stratified random sampling. Candidate predictors were subsequently selected using least absolute shrinkage and selection operator regression based exclusively on the training set, and multicollinearity was assessed using the variance inflation factor (VIF). For models requiring feature scaling, standardization parameters were estimated from the training data only and subsequently applied unchanged to the test data. Nine machine learning models were developed using the training set and evaluated in the independent test set. The final model was interpreted using SHAP, and an online prediction tool was developed based on important variables.

**Results:**

Among the 976 adults with ASD, 265 participants (27.2%) were classified as having moderate-to-severe insomnia. After LASSO selection using the training set, 18 predictors were retained, and no substantial multicollinearity was observed. In the held-out test set, logistic regression achieved a ROC AUC of 0.7262 and a PR AUC of 0.4624, compared with a no-skill precision-recall baseline of approximately 0.273. At the operating threshold used for classification, sensitivity was 0.4875, specificity was 0.8216, and accuracy was 0.7304, which was similar to the no-information rate of 0.7270. SHAP analysis showed that the total score of the non-sleep items of the GAD-9, the total score of the non-sleep items of the PHQ-8, anxiety diagnosis, proportion of screen time spent watching short videos, age, and sex were among the predictors with higher contributions.

**Conclusion:**

This study developed an interpretable prediction model for moderate-to-severe insomnia risk among adults with ASD. Anxiety- and depression-related symptoms were the main predictive features, while the proportion of screen time spent watching short videos also provided additional predictive value. The online tool developed based on key variables may provide an exploratory approach to individualized risk estimation and stratification, but further validation and threshold optimization are required before it can be considered for screening applications.

## 1 Introduction

Autism spectrum disorder (ASD) is a common neurodevelopmental condition characterized primarily by difficulties in social communication and social interaction, restricted or repetitive patterns of behavior, and atypical sensory processing (1, 2). In recent years, with increasing public awareness and improvements in screening and diagnostic practices, the identification rate of ASD has continued to rise, making it an important public health concern (3). According to 2022 surveillance data from the US Centers for Disease Control and Prevention, approximately 1 in 31 children aged 8 years has been identified with ASD; the World Health Organization similarly estimates that approximately 1 in 100 children worldwide has ASD (4, 5). As an increasing number of children with ASD transition into adulthood, the need for long-term health management among autistic adults has become increasingly prominent (6, 7). Beyond core symptoms, autistic adults frequently experience a range of psychiatric, psychological, behavioral, and physical health conditions, which may further affect daily functioning, social participation, quality of life, and healthcare needs (8, 9). Therefore, identifying health problems among autistic adults that are clinically actionable and of public health relevance is essential for improving their long-term health outcomes.

Sleep difficulties are common and persistent comorbid problems among individuals with ASD, typically manifesting as difficulty initiating sleep, difficulty maintaining sleep, poor sleep quality, early-morning awakening, and circadian rhythm disturbances (10, 11). Previous studies have shown that sleep problems in individuals with ASD are not limited to childhood and adolescence but may persist into adulthood (12). Among adults with ASD, sleep difficulties may be intertwined with anxiety, depression, daytime fatigue, reduced attention, difficulties in emotion regulation, and impaired social functioning, thereby further affecting education, employment, independent living, and quality of life (13, 14). From a public health perspective, long-term sleep problems may increase the burden of mental health conditions, healthcare utilization, and caregiving demands, while also reducing individuals’ ability to participate in social life and access sustained support (15, 16). Therefore, the early identification of clinically significant moderate-to-severe insomnia among adults with ASD may provide a basis for subsequent sleep assessment, psychological support, and integrated management.

Although sleep problems among individuals with ASD have received increasing attention, several limitations remain in the existing literature. First, previous studies have largely focused on children and adolescents with ASD, whereas sleep problems and their associated factors among adults with ASD remain relatively understudied. Adults with ASD are affected by multiple factors, including employment, independent living, social adaptation, co-occurring psychiatric and psychological conditions, and lifestyle changes; therefore, their risk of insomnia may differ from that observed in childhood and adolescence. Second, most existing studies have used traditional statistical methods and have primarily examined associations between sleep problems and individual or limited sets of risk factors, making it difficult to simultaneously integrate multidimensional information, including demographic characteristics, family and social factors, lifestyle behaviors, screen use, traumatic experiences, co-occurring diagnoses, and psychological and behavioral scales. Given the substantial heterogeneity of individuals with ASD, single factors are often insufficient to fully capture the risk of moderate-to-severe insomnia. Therefore, analytical approaches capable of integrating multisource variables are needed to more systematically predict and interpret the risk of moderate-to-severe insomnia among adults with ASD.

Machine learning approaches provide a promising framework for integrating multidimensional psychological and behavioral characteristics and enabling individualized risk prediction. Compared with traditional regression models, machine learning models can handle a larger number of candidate variables simultaneously and, to some extent, capture potentially complex relationships among variables. Accordingly, these approaches have been increasingly applied to risk prediction studies in mental health and sleep-related research (17, 18). Recent studies have further emphasized the importance of combining predictive modeling with explainability and practical digital implementation. Rony et al. developed an innovative ASD detection framework integrating feature selection, logistic regression, SHAP-based explainability, and a web application, providing a valuable example of how a concise set of informative features can be translated into an accessible digital tool. In the broader field of mental health, Ali et al. comprehensively summarized recent advances in artificial intelligence and highlighted interpretability, clinical relevance, and responsible implementation as important considerations for translating AI-based models into digital health practice (19, 20). However, the application of machine learning models in clinical and public health settings depends not only on predictive performance but also on model interpretability and translational potential. For identifying the risk of moderate-to-severe insomnia among adults with ASD, clinicians need not only to determine whether an individual is at high risk but also to understand which psychological, behavioral, or lifestyle factors contribute most substantially to risk prediction. The SHAP method can quantify the contribution of each variable to model output, providing an intuitive basis for interpreting model predictions and helping to reduce the practical limitations associated with “black-box” models.

Based on this background, the present study used psychological and behavioral survey data from adults with ASD to develop and validate a risk prediction model for moderate-to-severe insomnia. We first performed feature selection using least absolute shrinkage and selection operator regression and assessed multicollinearity among the selected variables using the variance inflation factor. We then compared the performance of multiple machine learning models, including logistic regression, XGBoost, random forest, LightGBM, and support vector machine, for predicting moderate-to-severe insomnia, and selected the model with the best overall performance for interpretability analysis. To enhance the clinical interpretability of the model, we further used SHAP to evaluate the contribution of each predictor to model output and explored differences in feature importance across subgroups defined by sex and physical activity level. Finally, informed by the explainability-guided and practical translation of predictive models described in recent research, we developed a simplified web-based prediction tool using a parsimonious subset of variables with the highest model contributions that are also readily obtainable in practice, with the aim of providing an accessible auxiliary tool for exploratory risk estimation and stratification of moderate-to-severe insomnia risk among adults with ASD.

## 2 Materials and methods

### 2.1 Study design and population

This study was conducted using data from the 2024 Psychological and Behavioral Survey Database of Adults with Autism Spectrum Disorder. This database was jointly established by Xiamen Xianyue Hospital, the Qingshan WeChat public account, and our research team, and was primarily designed to conduct an epidemiological survey of psychological and behavioral characteristics among adults with ASD through the WeChat platform. Eligible participants were adults with ASD who had been clinically diagnosed by psychiatrists and met the diagnostic criteria for typical autism, atypical autism, or Asperger syndrome, regardless of disease severity. Participants with secondary diagnoses such as depressive disorders, anxiety disorders, obsessive-compulsive disorder, or eating disorders were not excluded. All included participants were conscious, had the ability to communicate, were able to understand the informed consent information, and could complete the corresponding self-report scales according to their own conditions. Exclusion criteria included organic or symptomatic mental disorders, substance dependence, schizophrenia, bipolar disorder, or intellectual disability; current severe physical or neurological diseases; and insufficient reading ability in the participant or their primary caregiver to complete the assessment. After applying the inclusion and exclusion criteria and excluding cases with missing key variables, 976 adults with ASD were ultimately included in the analysis.

### 2.2 Outcome definition

The primary outcome was moderate-to-severe insomnia. Insomnia symptoms were assessed using the 7-item Insomnia Severity Index (ISI), a widely used self-report instrument for evaluating perceived insomnia severity. The total ISI score ranges from 0 to 28, with higher scores indicating more severe insomnia symptoms. According to standard scoring criteria, scores of 0–7 indicate no clinically significant insomnia, 8–14 indicate subthreshold insomnia, 15–21 indicate moderate clinical insomnia, and 22–28 indicate severe clinical insomnia. In this study, participants were classified into two groups according to the ISI total score. Those with ISI scores below 15 were classified as having no clinically significant or subthreshold insomnia, whereas those with ISI scores of 15 or higher were classified as having moderate-to-severe insomnia. Thus, the binary outcome variable was defined as moderate_severe_insomnia = 0 for ISI < 15 and moderate_severe_insomnia = 1 for ISI ≥ 15. This cutoff was selected to identify participants with clinically meaningful insomnia symptoms and has been commonly used in previous ISI-based research (21, 22).

### 2.3 Candidate predictor variables

Based on previous literature, clinical relevance, and data availability, this study included demographic characteristics, family and socioeconomic factors, lifestyle and screen-use behaviors, hobbies and interests, traumatic experiences, comorbid diagnoses, and scale scores as candidate predictors (23–25). Demographic, family, and social factors included sex, age, place of residence, educational level, paternal educational level, maternal educational level, family atmosphere, number of household cars, number of household computers, having an independent bedroom, and number of trips in the past year. Lifestyle and behavioral factors included body mass index, physical activity level, non-study screen time, proportion of screen time spent on gaming, proportion of screen time spent watching short videos, proportion of screen time spent chatting, having sports or exercise-related hobbies, absence of hobbies, and number of hobby categories. Physical activity level was classified according to moderate-to-vigorous physical activity as no moderate-to-vigorous physical activity, low physical activity, physical activity meeting recommended levels, and high physical activity. Variables related to traumatic experiences included exposure to any traumatic experience, number of trauma types, family abuse or neglect, school bullying, cyberbullying, and sexual assault. The clinical diagnostic variable included comorbid ADHD diagnosis. Psychological and behavioral scale variables included the total score of the non-sleep items of the PHQ-9, the total score of the non-sleep items of the GAD-10, the total score of the Chinese version of the Perceived Stress Scale, the total score of the Social Anxiety Scale for Adolescents, and the total score of the Chinese version of the Camouflaging Autistic Traits Questionnaire. To avoid outcome leakage, all ISI items and the ISI total score were excluded from the predictor set. In addition, sleep-related items were removed when calculating the total scores for depressive and anxiety symptoms to minimize direct overlap with the insomnia outcome.

### 2.4 Development and validation of machine learning models

The full dataset was first randomly divided into training and test sets at a ratio of 7:3 using stratified sampling to ensure a comparable proportion of moderate-to-severe insomnia outcomes in both sets. All subsequent feature-selection procedures were conducted using the training set only, and the test set was not used during feature selection or estimation of preprocessing parameters. Least absolute shrinkage and selection operator (LASSO) regression was subsequently performed exclusively in the training set to reduce the dimensionality of candidate variables and minimize the risk of model overfitting. The optimal penalization parameter λ in the LASSO model was determined using 10-fold cross-validation within the training set, and variables with non-zero coefficients corresponding to lambda.min were selected as features for subsequent model development (26). The selected variables were then examined using the variance inflation factor (VIF) to assess multicollinearity. VIF was used to determine whether substantial linear correlations existed among the variables included in the model and to inform subsequent model construction. Based on the selected variables, nine commonly used classification models were developed using the training set, including logistic regression, AdaBoost, XGBoost, support vector machine, random forest, LightGBM, gradient boosting, ExtraTrees, and multilayer perceptron. These models cover different algorithmic categories, including linear models, support vector machines, ensemble learning methods, gradient-boosted trees, and neural networks, allowing comparison of their ability to capture both linear and complex nonlinear relationships. For models sensitive to variable scales, including logistic regression, support vector machine, and multilayer perceptron, standardization parameters were estimated from the training data only and were subsequently applied unchanged to the test data. To address outcome class imbalance, imbalance-adjustment strategies were applied to selected algorithms where appropriate, including scale_pos_weight for XGBoost. No class weighting was applied to the logistic regression model. Hyperparameters for each model were optimized within the training set using 10-fold cross-validation and grid search. Model predictive performance was then evaluated in the independent test set. Finally, the optimal model was selected based on test-set ROC AUC, PR AUC, calibration performance, and model interpretability, and was subsequently used for SHAP-based interpretability analysis (27).

### 2.5 Statistical analysis

Statistical analyses were performed using R and Python. Continuous variables are presented as mean ± standard deviation, and categorical variables are presented as frequencies and percentages. Baseline characteristics between the training and test sets were compared using the chi-square test for categorical variables; Fisher’s exact test was used when expected cell counts were low, and the Wilcoxon rank-sum test was used for continuous variables. All statistical tests were two-sided, and P < 0.05 was considered statistically significant. To comprehensively evaluate the classification performance of different machine learning models, multiple performance metrics were used, including error rate, accuracy, F-beta score, area under the receiver operating characteristic curve (ROC AUC), sensitivity, specificity, area under the precision-recall curve (PR AUC), and Brier score. ROC curves were used to assess model discrimination, PR curves were used to evaluate predictive performance under class imbalance, and calibration curves were used to assess the agreement between predicted probabilities and observed outcomes. Decision curve analysis (DCA) was used to evaluate the net benefit of the models across different threshold probabilities and to assess their potential clinical utility. The stability of model performance was assessed using 10-fold cross-validation within the training set. The interpretability of the optimal model was examined using Shapley additive explanations (SHAP), which quantified the contribution of each predictor to the model output. SHAP beeswarm plots were used to display overall feature importance and the direction of each variable’s effect on prediction, while waterfall plots and force plots were used to illustrate individual-level predictive contributions. In addition, a Streamlit-based online risk prediction tool was developed based on the optimal model to provide individualized estimates of moderate-to-severe insomnia risk among adults with ASD.

## 3 Results

### 3.1 Baseline characteristics of the study population

A total of 976 adults with ASD were included in the final analysis, comprising 683 participants in the training set and 293 participants in the test set (**Table 1**). In the overall sample, 75.5% of participants were female, 91.8% lived in urban areas, and 71.2% had an undergraduate educational level. Most participants had an independent bedroom (84.3%); 81.9% reported at least one traumatic experience, and 47.5% had a comorbid diagnosis of ADHD. For continuous variables, the mean age was 24.39 ± 5.57 years, the mean BMI was 23.50 ± 7.22, and the mean non-study screen time was 8.01 ± 3.54 hours per day. No statistically significant differences were observed between the training and test sets for most demographic characteristics, family and socioeconomic factors, lifestyle behaviors, traumatic experiences, comorbid diagnoses, or psychological and behavioral scale scores (P > 0.05), suggesting that the two datasets were generally comparable. However, the training set had slightly higher PHQ-8 total scores without the sleep item than the test set (12.47 ± 5.22 vs. 11.77 ± 4.97, P = 0.047) and slightly higher GAD-9 total scores without the sleep item (16.32 ± 7.61 vs. 15.16 ± 7.48, P = 0.028).

**Table 1:** Baseline information of training set, test set.

Variable	Overall	Training set	Test set	P_Value
Gender	N=976	N=683	N=293	0.655
Male	239 (24.5%)	164 (24.0%)	75 (25.6%)	
Female	737 (75.5%)	519 (76.0%)	218 (74.4%)	
Residence	N=976	N=683	N=293	0.895
Rural	80 (8.2%)	57 (8.3%)	23 (7.8%)	
Urban	896 (91.8%)	626 (91.7%)	270 (92.2%)	
Education	N=976	N=683	N=293	0.728
High school or below	108 (11.1%)	72 (10.5%)	36 (12.3%)	
Undergraduate	695 (71.2%)	489 (71.6%)	206 (70.3%)	
Graduate or above	173 (17.7%)	122 (17.9%)	51 (17.4%)	
Father education	N=976	N=683	N=293	0.135
High school or below	568 (58.2%)	405 (59.3%)	163 (55.6%)	
College or bachelor	350 (35.9%)	233 (34.1%)	117 (39.9%)	
Graduate or above	58 (5.9%)	45 (6.6%)	13 (4.4%)	
Mother education	N=976	N=683	N=293	0.361
High school or below	622 (63.7%)	434 (63.5%)	188 (64.2%)	
College or bachelor	314 (32.2%)	217 (31.8%)	97 (33.1%)	
Graduate or above	40 (4.1%)	32 (4.7%)	8 (2.7%)	
Family atmosphere	N=976	N=683	N=293	0.244
Somewhat disharmonious	359 (36.8%)	255 (37.3%)	104 (35.5%)	
Very disharmonious	283 (29.0%)	199 (29.1%)	84 (28.7%)	
Somewhat harmonious	159 (16.3%)	117 (17.1%)	42 (14.3%)	
Very harmonious	175 (17.9%)	112 (16.4%)	63 (21.5%)	
Household car count	N=976	N=683	N=293	0.396
0	240 (24.6%)	168 (24.6%)	72 (24.6%)	
1	496 (50.8%)	355 (52.0%)	141 (48.1%)	
2	240 (24.6%)	160 (23.4%)	80 (27.3%)	
Household computer count	N=976	N=683	N=293	0.128
0	52 (5.3%)	32 (4.7%)	20 (6.8%)	
1	324 (33.2%)	225 (32.9%)	99 (33.8%)	
2	291 (29.8%)	196 (28.7%)	95 (32.4%)	
3	309 (31.7%)	230 (33.7%)	79 (27.0%)	
Own bedroom	N=976	N=683	N=293	0.783
No	153 (15.7%)	109 (16.0%)	44 (15.0%)	
Yes	823 (84.3%)	574 (84.0%)	249 (85.0%)	
Travel in past year	N=976	N=683	N=293	0.918
0	228 (23.4%)	162 (23.7%)	66 (22.5%)	
1	262 (26.8%)	179 (26.2%)	83 (28.3%)	
2	205 (21.0%)	144 (21.1%)	61 (20.8%)	
3	281 (28.8%)	198 (29.0%)	83 (28.3%)	
Physical activity level	N=976	N=683	N=293	0.891
No moderate/vigorous activity	553 (56.7%)	388 (56.8%)	165 (56.3%)	
Low activity	272 (27.9%)	187 (27.4%)	85 (29.0%)	
Recommended activity	90 (9.2%)	63 (9.2%)	27 (9.2%)	
High activity	61 (6.2%)	45 (6.6%)	16 (5.5%)	
Sports/exercise hobby	N=976	N=683	N=293	0.364
No	777 (79.6%)	538 (78.8%)	239 (81.6%)	
Yes	199 (20.4%)	145 (21.2%)	54 (18.4%)	
No hobby	N=976	N=683	N=293	0.703
No	934 (95.7%)	652 (95.5%)	282 (96.2%)	
Yes	42 (4.3%)	31 (4.5%)	11 (3.8%)	
Any traumatic experience	N=976	N=683	N=293	0.767
No	177 (18.1%)	126 (18.4%)	51 (17.4%)	
Yes	799 (81.9%)	557 (81.6%)	242 (82.6%)	
Family abuse or neglect	N=976	N=683	N=293	1.000
No	444 (45.5%)	311 (45.5%)	133 (45.4%)	
Yes	532 (54.5%)	372 (54.5%)	160 (54.6%)	
School bullying	N=976	N=683	N=293	0.297
No	416 (42.6%)	299 (43.8%)	117 (39.9%)	
Yes	560 (57.4%)	384 (56.2%)	176 (60.1%)	
Cyberbullying	N=976	N=683	N=293	0.942
No	870 (89.1%)	608 (89.0%)	262 (89.4%)	
Yes	106 (10.9%)	75 (11.0%)	31 (10.6%)	
Sexual assault	N=976	N=683	N=293	0.090
No	800 (82.0%)	550 (80.5%)	250 (85.3%)	
Yes	176 (18.0%)	133 (19.5%)	43 (14.7%)	
ADHD diagnosis	N=976	N=683	N=293	0.802
No	512 (52.5%)	356 (52.1%)	156 (53.2%)	
Yes	464 (47.5%)	327 (47.9%)	137 (46.8%)	
Age	24.39 ± 5.57	24.42 ± 5.48	24.30 ± 5.81	0.755
BMI	23.50 ± 7.22	23.26 ± 6.98	24.06 ± 7.74	0.129
Non-study screen time, hours/day	8.01 ± 3.54	8.03 ± 3.56	7.97 ± 3.48	0.815
Screen time: games, %	15.03 ± 19.25	14.78 ± 19.07	15.61 ± 19.67	0.542
Screen time: short video, %	15.84 ± 19.40	15.83 ± 19.33	15.85 ± 19.60	0.986
Screen time: chat, %	16.60 ± 16.37	16.01 ± 16.16	17.96 ± 16.80	0.094
Number of hobby categories	1.80 ± 1.16	1.81 ± 1.17	1.78 ± 1.16	0.743
Number of trauma types	1.80 ± 1.34	1.81 ± 1.37	1.77 ± 1.25	0.646
PHQ-8 total without sleep item	12.26 ± 5.15	12.47 ± 5.22	11.77 ± 4.97	0.047
GAD-9 total without sleep item	15.97 ± 7.58	16.32 ± 7.61	15.16 ± 7.48	0.028
CPSS total	34.34 ± 9.01	34.46 ± 8.92	34.04 ± 9.22	0.511
SAS-A total	64.18 ± 14.81	64.44 ± 14.88	63.56 ± 14.64	0.390
CAT-Q-Ch total	120.52 ± 23.62	120.38 ± 23.57	120.84 ± 23.75	0.779

### 3.2 Development and comparison of machine learning models

After the full dataset had been divided into training and test sets, LASSO regression was performed exclusively in the training set to select candidate predictors. As shown in **Figure 1**, as the penalization parameter λ changed, the coefficients of some variables were gradually shrunk to zero. Based on the optimal λ determined by 10-fold cross-validation within the training set, 18 variables with non-zero coefficients were ultimately retained for subsequent model development. Multicollinearity among the variables selected by LASSO was then assessed. The results showed that the VIF values of all selected variables were low, ranging from 1.0278 to 1.7459, and were within acceptable limits, indicating no substantial multicollinearity among the variables included in the models (**Supplementary Table 1**). Based on these selected variables, nine machine learning classification models were further developed and compared in terms of predictive performance (**Figures 2A–C**; **Supplementary Table 2**). Hyperparameters for each model were optimized within the training set using cross-validation and grid search, and the optimal parameter settings are shown in **Supplementary Table 3**. In the held-out test set, logistic regression achieved a ROC AUC of 0.7262 and a PR AUC of 0.4624. The PR AUC was higher than the no-skill baseline defined by the prevalence of moderate-to-severe insomnia in the test set (80/293, approximately 0.273), although the absolute improvement was modest. At the operating threshold used for classification, logistic regression had a sensitivity of 0.4875, specificity of 0.8216, balanced accuracy of 0.6545, PPV of 0.5065, NPV of 0.8102, and F1 score of 0.4968. Its overall accuracy was 0.7304, which was only marginally higher than the no-information rate of 0.7270 (**Supplementary Table 4**). The calibration curve showed some deviation between predicted and observed probabilities, particularly at higher predicted-risk levels (**Figure 2D**), indicating that absolute predicted risks should be interpreted cautiously. AdaBoost showed a higher sensitivity of 0.6875 but lower specificity (0.6667), reflecting a different sensitivity-specificity trade-off at the evaluated operating point. The ROC AUC values of XGBoost, support vector machine, random forest, LightGBM, and gradient boosting were all approximately 0.70, indicating broadly comparable overall performance. In contrast, ExtraTrees and multilayer perceptron showed relatively weaker discriminative ability. Overall, logistic regression showed modest discriminative performance while retaining advantages in model simplicity and interpretability. Its relatively low sensitivity at the evaluated operating point, however, indicates that the model should not be interpreted as a stand-alone screening classifier. Therefore, it was selected as the final model for subsequent model evaluation, SHAP-based interpretability analysis, and development of the online prediction tool.

**Figure 1:**
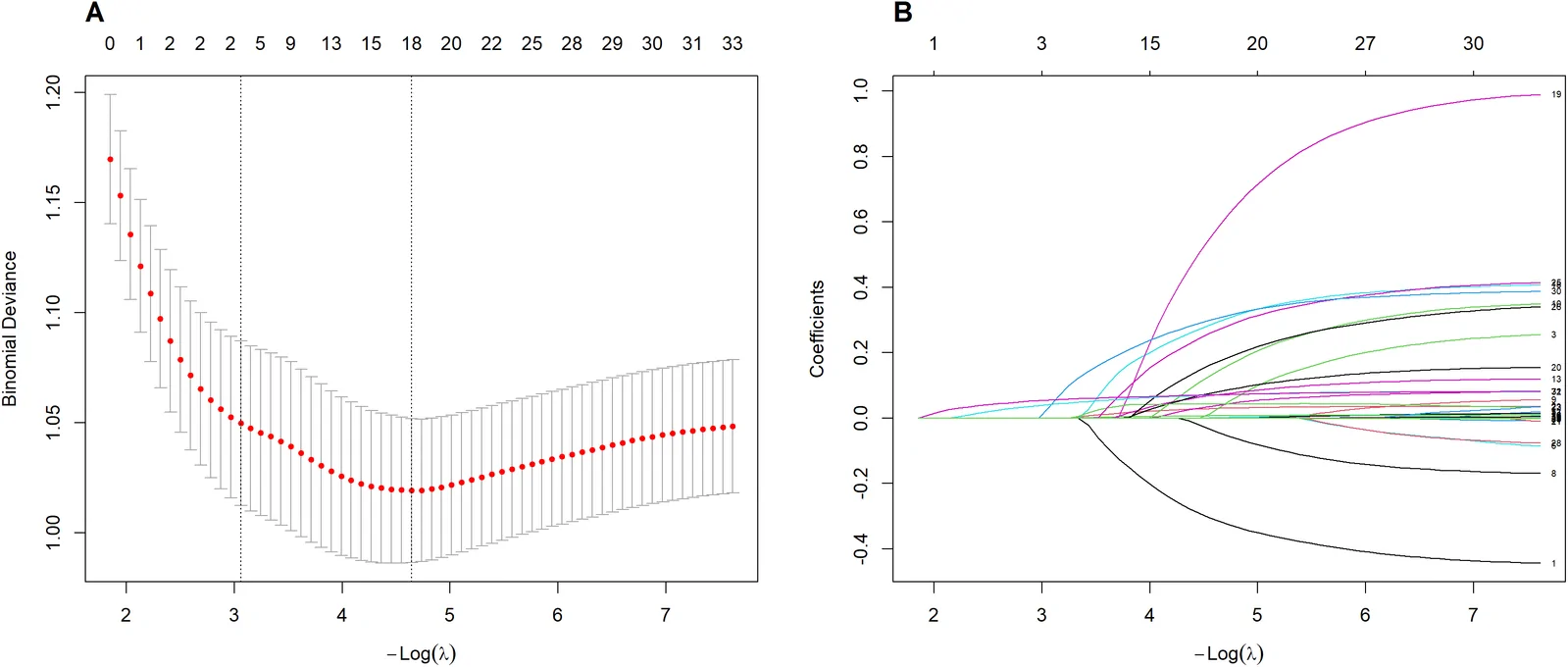
LASSO feature selection in the training set for predictors of moderate-to-severe insomnia in adults with ASD. **(A)** Ten-fold cross-validation within the training set for selecting the optimal penalty parameter lambda. **(B)** LASSO coefficient profiles of candidate predictors across different lambda values.

**Figure 2:**
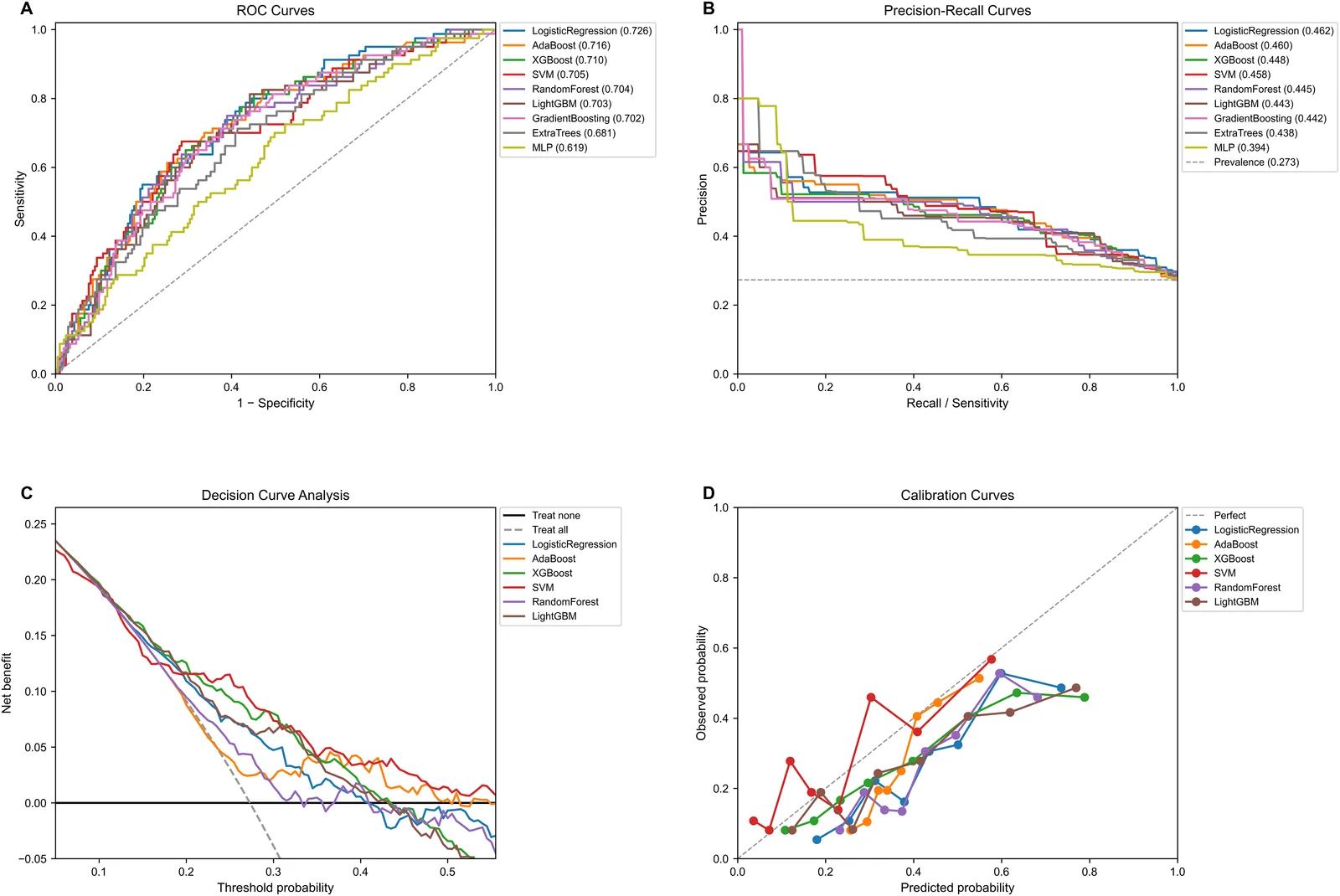
Performance comparison of machine learning models for predicting moderate-to-severe insomnia. **(A)** Receiver operating characteristic curves of different machine learning models. **(B)** Precision-recall curves of different machine learning models. **(C)** Decision curve analysis of selected machine learning models. **(D)** Calibration curves of selected machine learning models.

### 3.3 Model validation

To further evaluate the stability of model performance, 10-fold cross-validation was performed for each machine learning model. As shown in **Figure 3A**, the models showed differences in ROC AUC, PR AUC, accuracy, sensitivity, and specificity. Logistic regression showed relatively stable cross-validated ROC AUC and PR AUC performance, with a relatively stable distribution of results. Random forest, support vector machine, XGBoost, and AdaBoost showed comparable performance, whereas multilayer perceptron had lower ROC AUC, PR AUC, and sensitivity, suggesting relatively limited stability and predictive ability. Across individual cross-validation folds, logistic regression maintained relatively high ROC AUC values in most folds (**Figure 3B**). During repeated 10-fold cross-validation, the mean cross-validated ROC AUC of logistic regression remained within a relatively high range with limited variability, relatively stable internal validation performance. The heatmap results similarly showed that logistic regression, random forest, support vector machine, and XGBoost achieved higher ROC AUC values in most cross-validation folds, with logistic regression showing generally darker shading, indicating comparatively stronger overall validation performance (**Figure 3C**). Taken together, the cross-validation results further supported the selection of logistic regression as the final prediction model, although its performance beyond the present study population remains to be externally validated.

**Figure 3:**
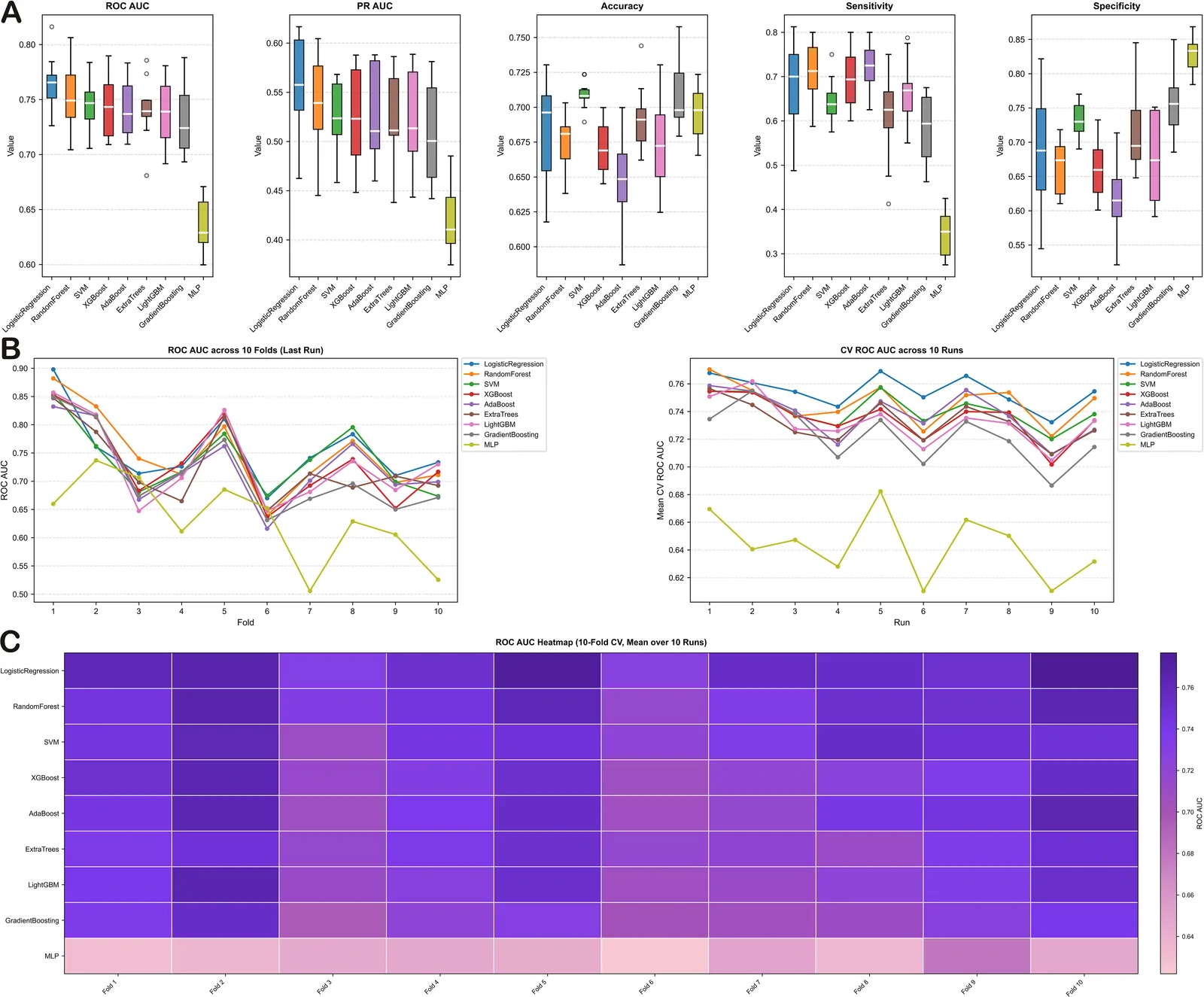
Cross-validation performance and stability of machine learning models. **(A)** Boxplots of cross-validation metrics across different machine learning models. **(B)** ROC AUC values across 10 folds and repeated cross-validation runs. **(C)** Heatmap of mean ROC AUC values from 10-fold cross-validation.

### 3.4 SHAP-based interpretation of feature importance in the final model

To further interpret the prediction mechanism of the final logistic regression model, SHAP was used to evaluate the contribution of each variable to the prediction of moderate-to-severe insomnia risk. According to the SHAP feature importance results, the total score of the non-sleep items of the GAD-9 made the largest contribution to the model output, with a mean absolute SHAP value of 0.3222. This was followed by the total score of the non-sleep items of the PHQ-8 (0.2621), anxiety diagnosis (0.2006), proportion of screen time spent watching short videos (0.1465), and age (0.1023) (**Supplementary Figure 1**). The SHAP beeswarm plot showed that individuals with higher GAD-9 and PHQ-8 scores generally had higher positive SHAP values, suggesting that elevated anxiety and depressive symptom levels were associated with higher model-predicted risk of moderate-to-severe insomnia. The proportion of screen time spent watching short videos, age, number of trauma types, and several hobby-related variables also contributed to the model output, although their overall contributions were lower than those of psychological symptom variables (**Figure 4A**). Individual-level waterfall and force plots further illustrated the prediction process for a single sample. In the example individual, higher GAD-9 score, higher PHQ-8 score, greater proportion of screen time spent watching short videos, and anxiety diagnosis all pushed the model prediction toward a higher risk and were the main factors contributing to the high-risk prediction. In contrast, several variables, such as number of trauma types and sports or exercise-related hobbies, made mild negative contributions to the predicted value (**Figures 4B, C**). Overall, the SHAP results suggested that anxiety and depressive symptoms were the most important contributors to the prediction of moderate-to-severe insomnia risk among adults with ASD, while screen-use behaviors and individual demographic characteristics also provided additional explanatory value. These SHAP values reflect contributions to model predictions and should not be interpreted as estimates of causal effects.

**Figure 4:**
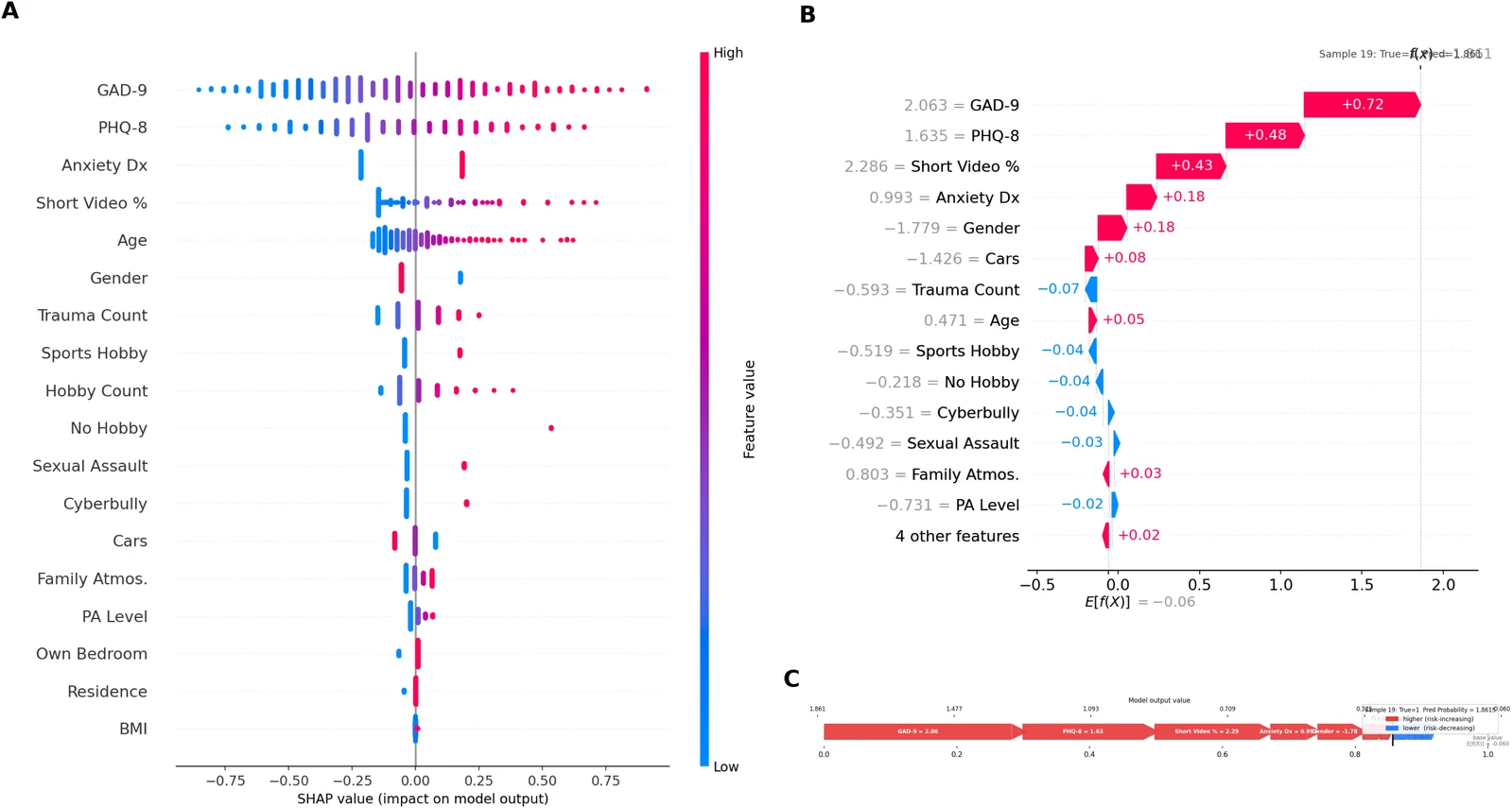
SHAP-based interpretation of the final logistic regression model. **(A)** SHAP beeswarm plot showing global feature contributions. **(B)** SHAP waterfall plot for an individual prediction example. **(C)** SHAP force plot for an individual prediction example.

### 3.5 Stratified SHAP analysis by sex and physical activity level

To further explore the consistency of model interpretability results across different subgroups, stratified SHAP analyses were performed according to sex and physical activity level. As shown in **Figure 5A**, GAD-9, PHQ-8, and anxiety diagnosis ranked among the top features in both male and female subgroups, suggesting that anxiety- and depression-related symptoms were core variables for predicting moderate-to-severe insomnia risk across sexes. In the male subgroup, the mean absolute SHAP values for GAD-9, PHQ-8, proportion of screen time spent watching short videos, age, number of hobby categories, and absence of hobbies were slightly higher than those in the female subgroup. By contrast, variables such as sexual assault, number of household cars, and having an independent bedroom showed relatively higher contributions in the female subgroup. After stratification by physical activity level, the ranking of major feature importance was generally consistent across subgroups (**Figure 5B**). GAD-9 and PHQ-8 consistently showed the highest contributions among participants with no moderate-to-vigorous physical activity, low physical activity, recommended levels of physical activity, and high physical activity, while anxiety diagnosis also remained among the leading features. Notably, the high physical activity subgroup showed the highest mean absolute SHAP value for GAD-9, whereas the no moderate-to-vigorous physical activity subgroup showed relatively higher contributions from the proportion of screen time spent watching short videos and absence of hobbies. Sports or exercise-related hobbies showed a higher contribution in the subgroup meeting recommended physical activity levels but a relatively lower contribution in the high physical activity subgroup. Overall, the stratified SHAP analyses showed that psychological symptom variables had stable and prominent predictive contributions across subgroups defined by sex and physical activity level. At the same time, the relative importance of factors such as screen use, hobbies and interests, and traumatic experiences varied across subgroups, suggesting that moderate-to-severe insomnia risk among adults with ASD may be jointly influenced by psychological, behavioral, and lifestyle-related factors.

**Figure 5:**
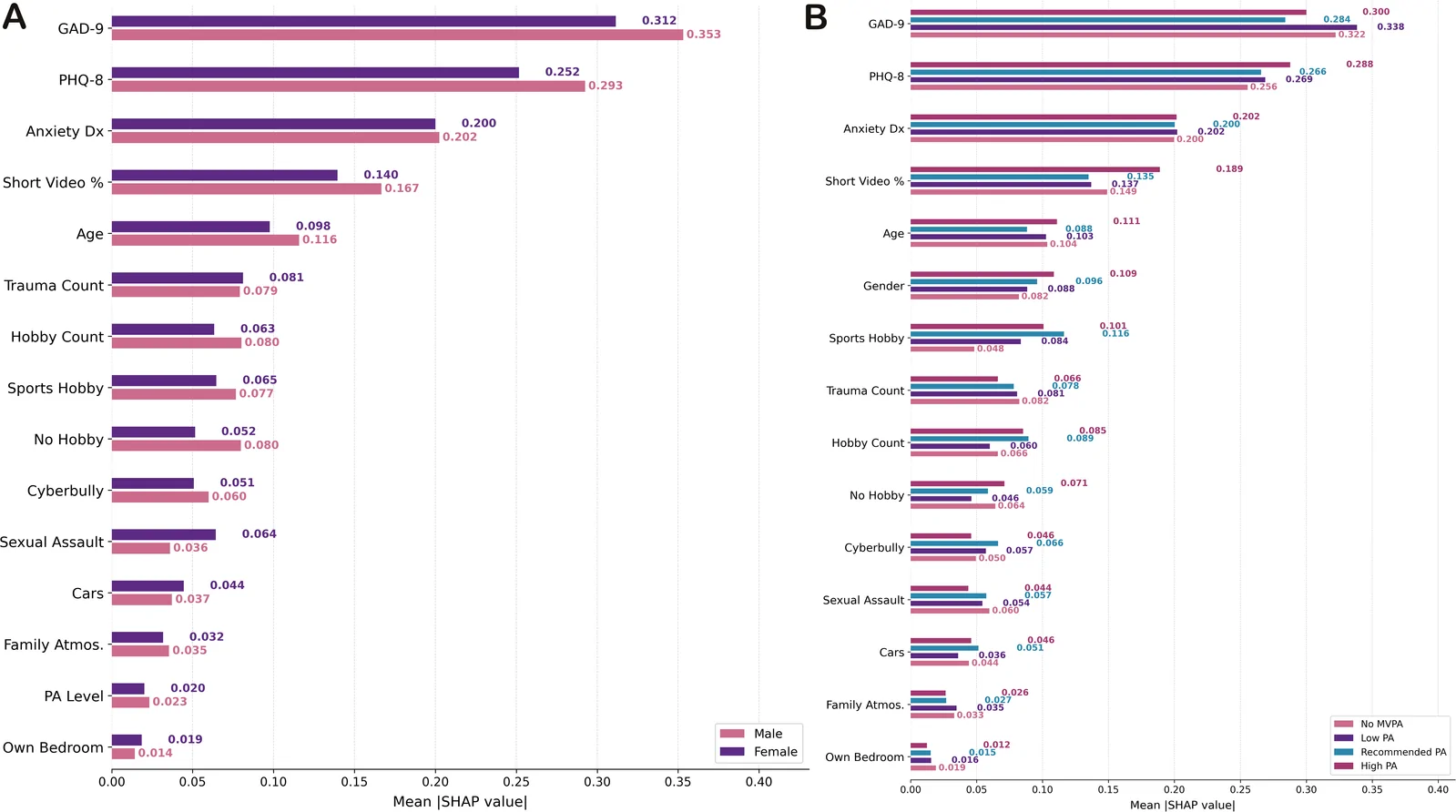
Stratified SHAP feature importance by sex and physical activity level. **(A)** SHAP feature importance stratified by sex. **(B)** SHAP feature importance stratified by physical activity level.

### 3.6 Development and potential clinical application of the online prediction tool

Based on the final logistic regression model and SHAP feature importance ranking, we developed a simplified six-input implementation of the final model as an online risk-estimation tool (https://asd-insomnia-predictor-26khfjyyobwetobvnzyihl.streamlit.app/). These predictors included the total score of the non-sleep items of the GAD-9, the total score of the non-sleep items of the PHQ-8, anxiety diagnosis, proportion of screen time spent watching short videos, age, and sex. For implementation, the remaining 12 predictors in the final model were fixed at their training-set median values, allowing users to enter only the six highest-ranking predictors while retaining the structure of the original model. The Streamlit-based tool is accessible through the URL provided above and allows users to enter individual-level information to obtain an estimated probability of moderate-to-severe insomnia. This online tool translates the model output into an accessible probability-based risk-estimation interface and is intended primarily for exploratory risk stratification rather than stand-alone screening or diagnosis. Given the limited sensitivity of the current model at the evaluated operating threshold, individuals should not be classified as free of clinically relevant insomnia solely on the basis of a low model-predicted risk. The tool may instead be used as an adjunct to clinical assessment, with predicted probabilities interpreted together with sleep-specific evaluation and individual clinical information. It is intended primarily for researchers and healthcare or community professionals; its performance has currently been evaluated internally only, without independent external or prospective clinical validation. Recent research on explainable AI in clinical decision support systems has highlighted that technical explainability alone does not ensure clinical usability, with clinician-centered evaluation and real-world integration remaining important translational challenges. No formal long-term clinical deployment or maintenance plan has yet been established; future studies should therefore evaluate usability, clinician acceptability, external validity, and prospective clinical performance before considering integration into routine practice (28).

## 4 Discussion

This study developed a risk prediction model for moderate-to-severe insomnia among adults with ASD and used interpretable machine learning methods to analyze the main predictive features. The results showed no substantial multicollinearity among the variables selected by LASSO. Among the nine machine learning models evaluated, logistic regression showed modest discriminative ability, with a test-set ROC AUC of 0.7262 and PR AUC of 0.4624, while retaining advantages in model simplicity and interpretability. However, its sensitivity of 0.4875 at the evaluated operating point indicates substantial limitations for binary screening. SHAP analysis indicated that the total score of the non-sleep items of the GAD-9, the total score of the non-sleep items of the PHQ-8, anxiety diagnosis, proportion of screen time spent watching short videos, age, and sex were among the variables with the highest contributions in the model. Stratified analyses further showed that anxiety- and depression-related symptoms maintained high importance across subgroups defined by sex and physical activity level. Overall, these findings suggest that psychological symptoms and certain lifestyle-related factors may serve as informative indicators for identifying the risk of moderate-to-severe insomnia among adults with ASD. The online tool developed using the top six variables should therefore be viewed primarily as an exploratory aid for probability-based risk estimation and stratification rather than as a validated screening instrument.

This study found that the total score of the non-sleep items of the GAD-9, the total score of the non-sleep items of the PHQ-8, and anxiety diagnosis were the predictors with the highest contributions in the model, which is generally consistent with previous findings on sleep problems among adults with ASD. Previous studies and systematic reviews have suggested that adults with ASD commonly experience poor sleep quality, prolonged sleep latency, and reduced sleep efficiency, and that sleep difficulties are often associated with anxiety, depression, stress levels, and reduced quality of life (29, 30). A systematic review and meta-analysis by Hollocks et al. on anxiety and depression in adults with ASD showed that anxiety and depression are common psychiatric comorbidities in this population (31). In the present study, sleep-related items were removed when calculating the PHQ-8 and GAD-9 scores; nevertheless, anxiety- and depression-related symptoms still carried the greatest weight in the model, indicating that this finding was not simply driven by overlap with sleep-related scale items. Compared with previous studies that primarily used correlation analyses, regression analyses, or between-group comparisons, the present study used machine learning and SHAP to quantify the predictive contribution of emotional symptoms to model-based identification of moderate-to-severe insomnia among adults with ASD. These findings should be interpreted as predictive associations rather than evidence that anxiety or depressive symptoms causally influence insomnia.

In addition to psychological symptoms, the proportion of screen time spent watching short videos also showed a relatively high SHAP contribution in the present model. Notably, this study did not use total non-study screen time alone, but rather the proportion of non-study screen time spent watching short videos. Therefore, this variable may reflect not only “how long individuals use screens” but also the structure of screen content and preferences in media use. Previous systematic reviews on electronic media and sleep have shown that electronic media use is associated with poorer sleep quality, delayed sleep onset, and shorter sleep duration (32). Recent studies on short-video use have also suggested that short-video use or short-video addiction is associated with poorer sleep quality, bedtime procrastination, and daytime sleepiness (33). Short-video platforms are typically characterized by rapid content switching, immediate rewards, algorithm-driven recommendations, and sustained engagement, which may more readily increase pre-sleep cognitive arousal, prolong use duration, and delay sleep onset (34). For some individuals with ASD, short-video use may not merely represent a form of entertainment, but may also serve functions related to emotion regulation, interest fulfillment, social substitution, or stress relief. Previous studies have shown that social media may provide adults with ASD with a relatively controllable social space, helping them maintain connections, find communities, and reduce the immediate pressure associated with face-to-face interactions (35). At the same time, technology-mediated activities may also be used for emotion regulation and the reduction of anxiety or stress. Therefore, the relationship between short-video use and insomnia risk may be more complex than that captured by traditional “screen time” indicators, involving multiple factors such as use duration, content type, usage motivation, and pre-sleep emotional state.

This study further conducted exploratory stratified SHAP analyses according to sex and physical activity level. The results showed that GAD-9 and PHQ-8 maintained high feature importance across subgroups defined by sex and physical activity level, suggesting that the contribution of emotional symptoms to model prediction was relatively stable. Meanwhile, several lifestyle-related variables showed different patterns of importance across subgroups. For example, the proportion of screen time spent watching short videos, age, and absence of hobbies showed relatively higher contributions in the male subgroup. Across physical activity subgroups, the contributions of the proportion of screen time spent watching short videos, sports or exercise-related hobbies, and absence of hobbies also varied. Previous studies have suggested that short-video use may be related to sleep health through pathways such as bedtime procrastination, cognitive arousal, and daytime fatigue, whereas physical activity and meaningful engagement in hobbies are also associated with sleep quality, mental health, and subjective well-being (36). Therefore, these findings suggest that screen use, activity level, and hobbies may jointly constitute the lifestyle context for identifying insomnia risk among adults with ASD. However, the observed subgroup differences should still be regarded as exploratory findings and used to inform future studies examining whether more complex joint effects exist among emotional symptoms, screen-use patterns, and lifestyle-related factors.

Based on the SHAP importance ranking, this study developed an online prediction tool using the top six variables, enabling the model results to be applied in practical assessment settings in a more simplified form. Compared with more complex models requiring complete variable input, this tool includes only readily obtainable information, including anxiety symptoms, depressive symptoms, anxiety diagnosis, proportion of screen time spent watching short videos, age, and sex, thereby reducing the barrier to use. Its potential value lies in helping clinicians or community service providers conduct preliminary risk stratification among adults with ASD and identify individuals who may require further sleep assessment or psychological support. However, this tool should be regarded as an auxiliary screening tool rather than a diagnostic instrument; its predictions should still be interpreted in combination with clinical interviews, scale-based assessments, and individual circumstances.

This study has several limitations. First, this was a cross-sectional observational study; therefore, the temporal sequence and causal relationships between psychological symptoms, screen-use behaviors, other predictors, and moderate-to-severe insomnia could not be determined. Accordingly, SHAP-derived feature importance should be interpreted as predictive contribution rather than causal effect. Second, the study data were primarily derived from self-report questionnaires, and the insomnia outcome was also classified based on the ISI scale, which may be subject to recall bias, subjective reporting bias, and common method bias. Third, both model development and evaluation were conducted within a single survey dataset, without an independent external validation cohort. In addition, because LASSO feature selection and model development were conducted within the same training dataset, some optimism in model performance and uncertainty in feature stability may remain despite evaluation in an independent held-out test set. Moreover, recruitment through a WeChat-based platform may have preferentially included individuals with greater internet access, engagement with online ASD communities, or healthcare-seeking behavior, potentially introducing selection bias. These factors, together with the relatively high proportion of female participants, may limit the representativeness of the sample and the generalizability of model performance to broader ASD populations and clinical settings. Independent validation in more diverse populations is therefore needed. Fourth, the model showed limited sensitivity at the evaluated operating threshold, restricting its use as a stand-alone screening instrument. Future studies should further examine threshold optimization according to the intended use.

## 5 Conclusions

This study developed a risk prediction model for moderate-to-severe insomnia among adults with ASD and interpreted the model using SHAP. The logistic regression model demonstrated modest discriminative ability, although sensitivity at the evaluated operating point was limited. Anxiety- and depression-related symptoms were the most important and relatively stable predictive features in the model, while the proportion of screen time spent watching short videos, age, and sex also provided additional information for risk identification. The online tool developed based on important variables may provide an exploratory aid for individualized risk estimation and stratification; however, further external validation and threshold optimization are required before its use for screening can be considered.

## Data Availability

The raw data supporting the conclusions of this article will be made available by the authors, without undue reservation.
