# Supplementary material for: Interpretable machine learning for predicting moderate-to-severe insomnia risk in Chinese adults with autism spectrum disorder
Source: Front Psychiatry. 2026 Sep 15;17:1898010. doi: 10.3389/fpsyt.2026.1898010 (PMC13619600; doi:10.3389/fpsyt.2026.1898010)
Supplement: Supplementary file 1 [file Table1.docx]

**Supplementary Table 1.** Variance Inflation Factor Analysis of LASSO-Selected Predictors.

| Variable | VIF | VIF_Flag |
| --- | --- | --- |
| phq8_no_sleep_total | 1.745905 | Acceptable |
| trauma_count | 1.74542 | Acceptable |
| gad9_no_sleep_item5_total | 1.74528 | Acceptable |
| trauma_sexual_assault | 1.396022 | Acceptable |
| hobby_sports_exercise | 1.380994 | Acceptable |
| hobby_category_count | 1.380457 | Acceptable |
| trauma_cyberbullying | 1.311492 | Acceptable |
| physical_activity_level | 1.248788 | Acceptable |
| hobby_no_hobby | 1.181145 | Acceptable |
| age | 1.087704 | Acceptable |
| gender | 1.076174 | Acceptable |
| diagnosis_anxiety | 1.074914 | Acceptable |
| household_car_count | 1.073026 | Acceptable |
| own_bedroom | 1.055146 | Acceptable |
| screen_short_video_pct | 1.050223 | Acceptable |
| residence | 1.043516 | Acceptable |
| family_atmosphere | 1.028572 | Acceptable |
| bmi | 1.027787 | Acceptable |
