# Supplementary material for: Interpretable machine learning for predicting moderate-to-severe insomnia risk in Chinese adults with autism spectrum disorder
Source: Front Psychiatry. 2026 Sep 15;17:1898010. doi: 10.3389/fpsyt.2026.1898010 (PMC13619600; doi:10.3389/fpsyt.2026.1898010)
Supplement: Supplementary file 2 [file Table2.docx]

**Supplementary Table 2.** Performance comparison of different machine learning models.

| Model | Threshold | Accuracy | Error Rate | F-beta (beta=2) | ROC AUC | Sensitivity | Specificity | PR AUC | Brier Score | CV ROC AUC |
| --- | --- | --- | --- | --- | --- | --- | --- | --- | --- | --- |
| LogisticRegression | 0.5287 | 0.7304 | 0.2696 | 0.4912 | 0.7262 | 0.4875 | 0.8216 | 0.4624 | 0.1983 | 0.7676 |
| AdaBoost | 0.3742 | 0.6724 | 0.3276 | 0.6166 | 0.7161 | 0.6875 | 0.6667 | 0.4599 | 0.191 | 0.7599 |
| XGBoost | 0.4688 | 0.6894 | 0.3106 | 0.58 | 0.7102 | 0.625 | 0.7136 | 0.4483 | 0.1992 | 0.755 |
| SVM | 0.2975 | 0.7065 | 0.2935 | 0.5502 | 0.7055 | 0.575 | 0.7559 | 0.4583 | 0.1802 | 0.7518 |
| RandomForest | 0.4644 | 0.6826 | 0.3174 | 0.5504 | 0.7043 | 0.5875 | 0.7183 | 0.4452 | 0.2022 | 0.7674 |
| LightGBM | 0.5121 | 0.6962 | 0.3038 | 0.5276 | 0.7029 | 0.55 | 0.7512 | 0.4434 | 0.2016 | 0.7533 |
| GradientBoosting | 0.265 | 0.6826 | 0.3174 | 0.5504 | 0.702 | 0.5875 | 0.7183 | 0.4419 | 0.1873 | 0.7447 |
| ExtraTrees | 0.5309 | 0.6962 | 0.3038 | 0.4177 | 0.6809 | 0.4125 | 0.8028 | 0.438 | 0.2073 | 0.7529 |
| MLP | 0.6532 | 0.6997 | 0.3003 | 0.2957 | 0.6193 | 0.275 | 0.8592 | 0.3937 | 0.2883 | 0.6638 |
