# Supplementary material for: Interpretable machine learning for predicting moderate-to-severe insomnia risk in Chinese adults with autism spectrum disorder
Source: Front Psychiatry. 2026 Sep 15;17:1898010. doi: 10.3389/fpsyt.2026.1898010 (PMC13619600; doi:10.3389/fpsyt.2026.1898010)
Supplement: Supplementary file 3 [file Table3.docx]

**Supplementary Table 3.** Best Hyperparameter Settings for Each Machine Learning Model.

| Model | Best Params |
| --- | --- |
| LogisticRegression | {'clf__C': 0.01, 'clf__penalty': 'l2'} |
| AdaBoost | {'clf__learning_rate': 0.5, 'clf__n_estimators': 100} |
| XGBoost | {'clf__colsample_bytree': 1.0, 'clf__learning_rate': 0.03, 'clf__max_depth': 2, 'clf__n_estimators': 200, 'clf__subsample': 0.8} |
| SVM | {'clf__C': 0.1, 'clf__kernel': 'rbf'} |
| RandomForest | {'clf__max_depth': 3, 'clf__min_samples_leaf': 5, 'clf__n_estimators': 400} |
| LightGBM | {'clf__learning_rate': 0.03, 'clf__max_depth': 2, 'clf__n_estimators': 200, 'clf__num_leaves': 7} |
| GradientBoosting | {'clf__learning_rate': 0.05, 'clf__max_depth': 2, 'clf__n_estimators': 200, 'clf__subsample': 1.0} |
| ExtraTrees | {'clf__max_depth': 5, 'clf__n_estimators': 200} |
| MLP | {'clf__alpha': 0.01, 'clf__hidden_layer_sizes': (32, 16)} |
