# Supplementary material for: Interpretable machine learning for predicting moderate-to-severe insomnia risk in Chinese adults with autism spectrum disorder
Source: Front Psychiatry. 2026 Sep 15;17:1898010. doi: 10.3389/fpsyt.2026.1898010 (PMC13619600; doi:10.3389/fpsyt.2026.1898010)
Supplement: Supplementary file 4 [file Table4.docx]

**Supplementary Table 4.** Confusion matrix-derived classification metrics for candidate machine-learning models in the held-out test set.

| Model | TP | FP | TN | FN | Accuracy | NIR | Sensitivity | Specificity | Balanced  Accuracy | PPV | NPV | F1 |
| --- | --- | --- | --- | --- | --- | --- | --- | --- | --- | --- | --- | --- |
| Logistic Regression | 39 | 38 | 175 | 41 | 0.7304 | 0.727 | 0.4875 | 0.8216 | 0.6545 | 0.5065 | 0.8102 | 0.4968 |
| AdaBoost | 55 | 71 | 142 | 25 | 0.6724 | 0.727 | 0.6875 | 0.6667 | 0.6771 | 0.4365 | 0.8503 | 0.534 |
| XGBoost | 50 | 61 | 152 | 30 | 0.6894 | 0.727 | 0.625 | 0.7136 | 0.6693 | 0.4505 | 0.8352 | 0.5236 |
| SVM | 46 | 52 | 161 | 34 | 0.7065 | 0.727 | 0.575 | 0.7559 | 0.6654 | 0.4694 | 0.8256 | 0.5169 |
| Random Forest | 47 | 60 | 153 | 33 | 0.6826 | 0.727 | 0.5875 | 0.7183 | 0.6529 | 0.4393 | 0.8226 | 0.5027 |
| LightGBM | 44 | 53 | 160 | 36 | 0.6962 | 0.727 | 0.55 | 0.7512 | 0.6506 | 0.4536 | 0.8163 | 0.4972 |
| Gradient Boosting | 47 | 60 | 153 | 33 | 0.6826 | 0.727 | 0.5875 | 0.7183 | 0.6529 | 0.4393 | 0.8226 | 0.5027 |
| Extra Trees | 33 | 42 | 171 | 47 | 0.6962 | 0.727 | 0.4125 | 0.8028 | 0.6077 | 0.44 | 0.7844 | 0.4258 |
| MLP | 22 | 30 | 183 | 58 | 0.6997 | 0.727 | 0.275 | 0.8592 | 0.5671 | 0.4231 | 0.7593 | 0.3333 |

NIR, no-information rate; PPV, positive predictive value; NPV, negative predictive value. The no-information rate was calculated as the proportion of the majority class in the held-out test set.
